# *QuickStats:* Percentage* of Adults Aged ≥18 Years with Chronic Pain in the Past 3 Months,^†^ by Sex and Urbanization Level^§^ — United States, 2023

**DOI:** 10.15585/mmwr.mm7407a5

**Published:** 2025-03-06

**Figure:**
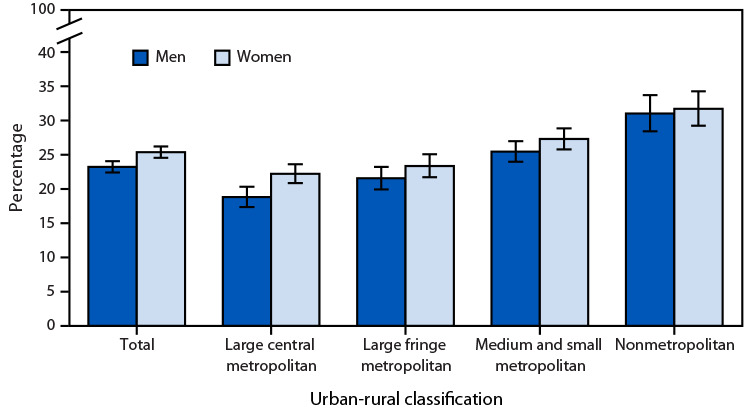
In 2023, the percentage of adults aged ≥18 years with chronic pain in the past 3 months was higher among women (25.4%) than among men (23.2%) overall. A higher percentage of women than men in large central metropolitan areas experienced chronic pain (22.2% versus 18.8%, respectively); differences for the other urbanization levels were not significant. Among both men and women, prevalence of recent chronic pain increased with decreasing urbanicity.

**Supplementary Table:** https://stacks.cdc.gov/view/cdc/176063

**Source:** National Center for Health Statistics, National Health Interview Survey, 2023. https://www.cdc.gov/nchs/nhis.htm

